# Regulation of ethanol intake under chronic mild stress: roles of dopamine receptors and transporters

**DOI:** 10.3389/fnbeh.2015.00118

**Published:** 2015-05-12

**Authors:** Foteini Delis, Christina Rombola, Robert Bellezza, Lauren Rosko, David K. Grandy, Nora D. Volkow, Panayotis K. Thanos

**Affiliations:** ^1^Department of Psychology, State University of New York at Stony BrookStony Brook, NY, USA; ^2^Department of Physiology & Pharmacology, School of Medicine, Oregon Health & Science UniversityPortland, OR, USA; ^3^Laboratory of Neuroimaging, National Institute on Alcohol Abuse and AlcoholismBethesda, MD, USA

**Keywords:** dopamine transporter, D2 receptor, D1 receptor, chronic mild stress, ethanol

## Abstract

Studies have shown that exposure to chronic mild stress decreases ethanol intake and preference in dopamine D2 receptor wild-type mice (*Drd2*^+/+^), while it increases intake in heterozygous (*Drd2*^+/−^) and knockout (*Drd2*^−/−^) mice. Dopaminergic neurotransmission in the basal forebrain plays a major role in the reinforcing actions of ethanol as well as in brain responses to stress. In order to identify neurochemical changes associated with the regulation of ethanol intake, we used *in vitro* receptor autoradiography to measure the levels and distribution of dopamine D1 and D2 receptors and dopamine transporters (DAT). Receptor levels were measured in the basal forebrain of *Drd2*^+/+^, *Drd2*^+/−^, and *Drd2*^−/−^ mice belonging to one of four groups: control (C), ethanol intake (E), chronic mild stress exposure (S), and ethanol intake under chronic mild stress (ES). D2 receptor levels were higher in the lateral and medial striatum of *Drd2*^+/+^ ES mice, compared with *Drd2*^+/+^ E mice. Ethanol intake in *Drd2*^+/+^ mice was negatively correlated with striatal D2 receptor levels. D2 receptor levels in *Drd2^+/−^* mice were the same among the four treatment groups. DAT levels were lower in *Drd2^+/−^ C* and *Drd2*^−/−^ C mice, compared with *Drd2*^+/+^ C mice. Among *Drd2^+/−^ mice*, S and ES groups had higher DAT levels compared with C and E groups in most regions examined. In Drd2^−/−^ mice, ethanol intake was positively correlated with DAT levels in all regions studied. D1 receptor levels were lower in Drd2^+/−^ and Drd2^−/−^ mice, compared with Drd2^+/+^, in all regions examined and remained unaffected by all treatments. The results suggest that in normal mice, ethanol intake is associated with D2 receptor-mediated neurotransmission, which exerts a protective effect against ethanol overconsumption under stress. In mice with low *Drd2* expression, where DRD2 levels are not further modulated, ethanol intake is associated with DAT function which is upregulated under stress leading to ethanol overconsumption.

## Introduction

Research shows that alcohol addiction has a strong genetic component shaped by many genes (for review see Enoch, 2014). *Drd2* encodes for the dopamine D2 receptor protein and is one of these genes with a strong regulatory role on alcohol intake. *In vivo*, *in vitro*, preclinical, and animal studies have shown that alcoholism and ethanol (ETOH) consumption are negatively modulated by dopamine D2 receptors (Blum et al., 1990; Stefanini et al., 1992; McBride et al., 1993; Volkow et al., 1996, 2006; Thanos et al., 2001, 2005; Tupala et al., 2001). Due to the comorbid nature of the disease, alcohol-related studies often investigate the role of stress in the development of alcoholism either through direct, and reciprocal, interactions between stress experience and levels of alcohol intake (Anthenelli and Grandison, 2012) or through more complex interactions among stress, ETOH consumption, and specific genetic factors (Anthenelli, 2012).

We have previously shown that when normal, *Drd2*^+/+^, mice are exposed to chronic mild stress (CMS) they decrease their ETOH intake and preference. In contrast, *Drd2*^+/−^ and *Drd2*^−/−^ mice exposed to the same CMS protocol increase their ETOH intake and preference (Delis et al., 2013). Here we sought to study responses of the brain dopamine system relating to CMS and ETOH intake, as a function of *Drd2* expression. To this purpose, we studied dopamine D1 and D2 receptor and dopamine transporter (DAT) levels in the basal forebrain of *Drd2*^+/+^, *Drd2*^+/−^, and *Drd2*^−/−^ mice that were exposed to CMS, ETOH, or their combination. Our study showed that lower ETOH intake under CMS in *Drd2*^+/+^ mice was associated with increased D2 receptor levels in the striatum, in agreement with previous findings. In contrast, higher ETOH intake under CMS in *Drd2*^+/−^ and *Drd2*^−/−^ mice was associated with higher DAT levels in the striatum and the n. accumbens.

## Materials and methods

### Animals

Eighty-six male *Drd2*^+/+^, *Drd2*^+/−^, and *Drd2*^−/−^ mice were used in this study. The mice were originally obtained from the laboratory of Dr. David Grandy and bred accordingly as previously described (Kelly et al., 1998) from *Drd2*^+/−^ breeders congenic on C57Bl6 strain. After weaning, the mice were tattooed on the tail and a 1 mm long tailsnip was also obtained and used for genotyping (Transnetyx, Cordova, TN). All animals were individually housed and kept on a 12:12 L/D reverse cycle with lights off at 07:00. Food and water were provided *ad lib*. The Institutional Animal Care and Use Committee (IACUC) of Stony Brook University approved this work in accordance with the guidelines established by the National Institutes of Health in “The Guide for Care and Use of Laboratory Animals.”

### Chronic mild stress and ethanol treatments

The animals were randomly assigned to one of the four following groups: control, chronic mild stress (CMS) only, ETOH only, CMS + ETOH, as described in detail in our previous publication (Delis et al., 2013). The CMS protocol was adapted from previous studies (Muscat et al., 1992) but did not include food and water deprivation. All mice had access to two bottles of water starting 1 week prior to the beginning of the behavioral experiments. Mice in the two groups that were given ETOH (E and ES) had continuous access to both 5% (v/v) ETOH and water starting from the beginning of Week 2. Mice not in an ETOH group continued to have access to two bottles of water throughout the study. The position of the bottles was switched daily to prevent a position preference bias.

### *In vitro* receptor autoradiography

Twenty four hours after the end of the experiment, between 09:00 and 12:00, mice were anesthetized with isoflurane and decapitated; brains were rapidly extracted, flash-frozen in methyl butane, and stored at −80°C. Fifteen micrometer-thick coronal brain sections were cut with the use of a cryostat, thaw-mounted on glass slides, and stored at −20°C in tightly sealed slide boxes until the day of the receptor binding experiment.

Dopamine Transporter (DAT) binding was assayed according to a previously established protocol (Hebert et al., 1999) using 3.5 nM [^3^H]WIN35428 (Specific activity 64Ci/mmol, PerkinElmer, Waltham, MA) as the radioligand. Non-specific binding was determined in the presence of 30 μM cocaine (Sigma-Aldrich, USA). The total sample size was 86, with 6–9 mice per group.

D1 dopamine receptor binding was performed according to a previously established protocol (Tarazi et al., 1997) using 2.5 nM [^3^H]SCH 23390 (Specific activity 85Ci/mmol, PerkinElmer, Waltham, MA) and 40 nM ketanserin to block 5HT2a binding sites. Non-specific binding was determined in the presence of 1 μM cis-flupenthixol (Sigma Aldrich, USA). The total sample size was 81, with 6–8 mice per group. Slides or sections from 5 mice were damaged during the assay and were not analyzed.

D2 dopamine receptor binding was performed according to a previously established protocol (Tarazi et al., 1997) using 2 nM [^3^H]raclopride (70Ci/mmol, PerkinElmer, Waltham, MA). Non-specific binding was determined in the presence of 10 μM sulpiride (Sigma Aldrich, USA). Binding was performed on tissue from *Drd2*^+/+^ and *Drd2*^+/−^ mice and the total sample size was 60, with 6–7 mice per group.

### Quantification

The slides were exposed to ^3^H-sensitive film (BiomaxMR, Kodak, USA) for 4–12 weeks along with [^3^H] microscales (ARC, St. Louis, MO, USA). The films were developed in Kodak D19 developer and scanned under constant conditions. Optical densities and bound radioactivity were measured with Image J software. The main body of the striatum was divided in quadrants [dorsolateral (DL), dorsomedial (DM), ventrolateral (VL), ventromedial (VM)], and the caudal striatal sections (the tail of the striatum) in two parts (dorsal, ventral).

### Statistical analysis

Receptor autoradiography measurements were analyzed using a three-way ANOVA, with Genotype (3 levels for DAT and D1 binding; 2 levels for D2 binding), Ethanol (2 levels, H_2_O/ETOH) and Stress (2 levels, NO/CMS) as between-subjects factors. When appropriate, the Tukey *post-hoc* test was applied to determine the significant pairwise differences. Overall threshold of significance was set at *p* < 0.05. All data are presented as mean ± standard error of the mean.

## Results

### Ethanol intake

We previously showed that CMS decreased ETOH intake and preference in *Drd2*^+/+^ mice and increased it in *Drd2*^+/−^ and *Drd2*^−/−^ mice (Delis et al., 2013). These results are presented in Table 1 to help the reader follow the results and discussion of the current study. CMS-induced decrease in *Drd2*^+/+^ ETOH intake/preference was driven by their lower ETOH consumption, while the respective increases in *Drd2*^+/−^ and *Drd2*^−/−^ were driven by their higher ETOH consumption as well as their lower water intake (Table 1) (Two-way ANOVA for water intake, with Genotype and Stress as between subjects factors: Genotype × Stress *F*_(2, 74)_ = 3.22, *p* = 0.041, ***Drd2***^+/+^: No Stress vs. CMS *p* = 0.99, ***Drd2***^+/−^: No Stress vs. CMS *p* = 0.011, ***Drd2***^−/−^: No Stress vs. CMS *p* = 0.041). Body weights and total liquid intake were not affected by the treatments.

**Table 1:** **Ethanol intake and preference modulated by chronic mild stress and *Drd2* expression**.

		***Drd2***+/+	***Drd2***+/−	***Drd2***−/−
Average %	E	52.9 ± 2.5	41.6 ± 2.9^∧^	32.9 ± 2.4^∧^
preference	SE	45.7 ± 1.2^*^	55.6 ± 2.3^*^^∧^	47.1 ± 3.6^*^
Total ETOH intake	E	95.2 ± 4.5	65.6 ± 5.7^∧^	53.6 ± 6.7^∧^
(g ETOH/kg b.w.)	SE	63.8 ± 4.5^*^	86.2 ± 7.8^*^^∧^	77.2 ± 6.9^*^
Total ETOH solution intake	E	2408 ± 143	1663 ± 144^∧^	1359 ± 168^∧^
(g solution/kg b.w.)	SE	1618 ± 114^*^	2185 ± 224^*^^∧^	1960 ± 175^*^
Total H_2_O intake from H_2_O bottle	E	2110 ± 210	2533 ± 182	2797 ± 302
(g H_2_O/kg b.w.)	SE	2108 ± 106	1600 ± 127^*^	1983 ± 259^*^
	C	4330 ± 277	3773 ± 119	4073 ± 241
Total liquid intake	E	4518 ± 276	4196 ± 297	4156 ± 452
(g/kg b.w.)	S	4261 ± 241	3800 ± 193	4220 ± 206
	SE	3726 ± 153	3785 ± 300	3943 ± 337
	C	27.4 ± 0.7	27.2 ± 0.4	28.1 ± 0.6
Body weight (g)	E	28.7 ± 1.0	27.0 ± 0.3	27.9 ± 0.5
	S	28.9 ± 0.5	28.2 ± 0.7	27.6 ± 0.6
	SE	28.3 ± 1.0	28.0 ± 0.5	28.6 ± 0.6

*C, control; E, ETOH 2 bottle choice without CMS; S, CMS exposure; SE, ETOH 2 bottle choice with CMS; Average % preference was calculated as average daily [ETOH solution consumed (g)/total liquid consumption (g)]; “Total H_2_O intake from H_2_O bottle” corresponds to intake during weeks 2–4, in order to be compared to “ETOH solution intake” during the same period. “Total liquid intake” corresponds to liquid intake from the 2 bottles during weeks 2–4. “Body weight” corresponds to average body weight during weeks 2–4. b.w.: body weight*.

* *Compared with E*,

∧ *compared with Drd2^+/+^*.

### DAT binding

**Figure 1:**
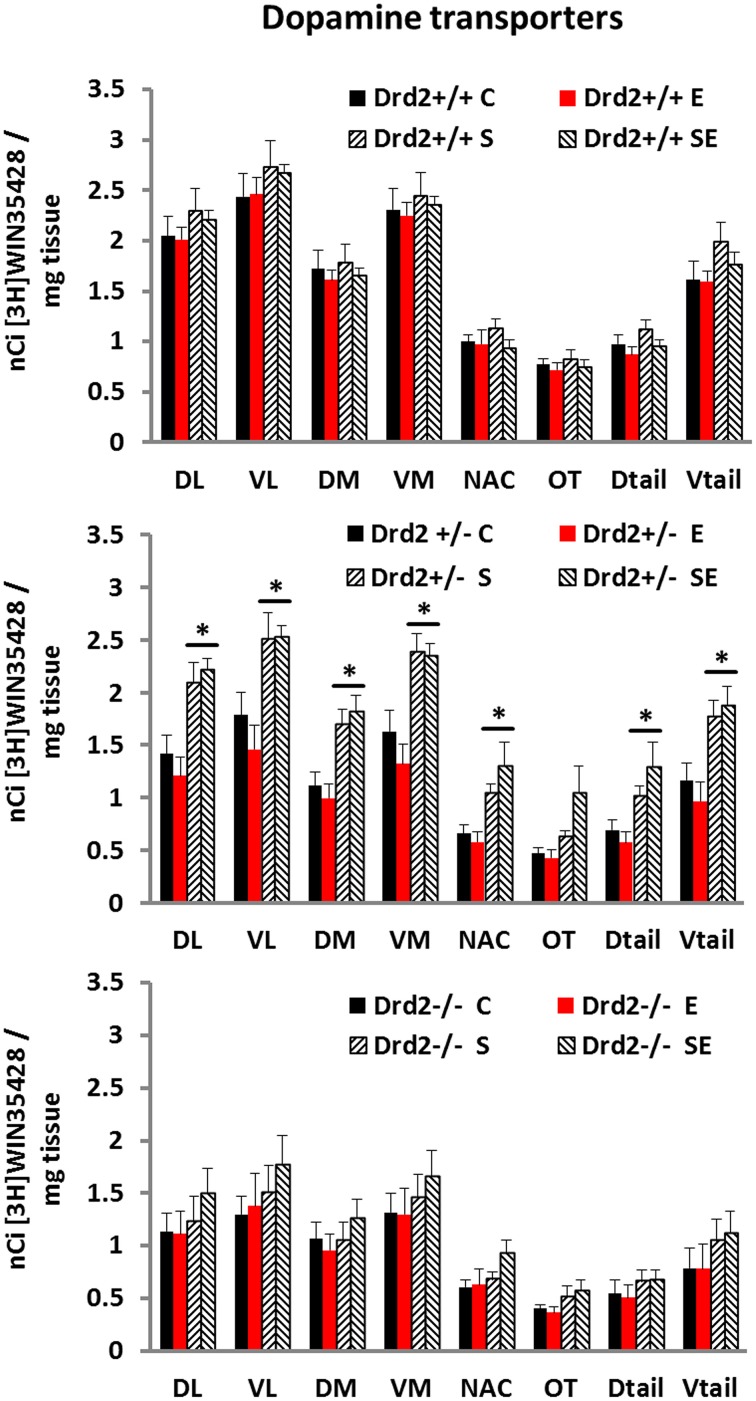
**Specific [^3^H]WIN35428 binding in the basal forebrain of *Drd2*^+/+^, *Drd2*^+/−^, and*Drd2*^−/−^mice**. The bars represent mean + S.E.M., C, control; E, ethanol; S, chronic mild stress (CMS); SE CMS + ETOH; ^*^compared with C and E; *Drd2*^−/−^ and non-stressed *Drd2*^+/−^ measures are significantly different from the respective *Drd2*^+/+^ measures (for details please see Results).

We performed an *in vitro* quantitative autoradiographic study of [^3^H]WIN35428 specific binding to determine changes in the levels and distribution of dopamine transporters associated with *Drd2* expression, with CMS exposure, and with ETOH intake (Figure 1). Three-way ANOVA for [^3^H]WIN35428 specific binding showed significant main effects of Genotype and Stress and a significant Genotype × Stress interaction in most regions studied. *Post-hoc* pairwise comparisons with Tukey test showed that non-stressed (C and E) *Drd2*^+/+^ mice had significantly higher DAT binding levels compared with non-stressed *Drd2*^+/−^ and non-stressed *Drd2*^−/−^ mice in the four quadrants of the striatum. The results also showed that stressed *Drd2*^+/−^ mice (S and SE) had significantly higher DAT binding compared with non-stressed mice (C and E) of the same genotype in the four quadrants and the tail of the striatum and in the n. accumbens (**DL striatum**: Genotype *F*_(2, 74)_ = 20.99, *p* < 0.001, Stress *F*_(1, 74)_ = 17.86, *p* < 0.001, Genotype × Stress *F*_(2, 74)_ = 3.08, *p* = 0.041; **No Stress**: *Drd2*^+/+^ vs. *Drd2*^+/−^*p* = 0.012, *Drd2*^+/+^ vs. *Drd2*^−/−^*p* = 0.002; **CMS**: *Drd2*^+/+^ vs. *Drd2*^−/−^*p* < 0.001, *Drd2*^+/−^ vs. *Drd2*^−/−^*p* = 0.001; ***Drd2***^+/−^: CMS vs. No Stress: *p* < 0.001. **VL striatum**: Genotype *F*_(2, 74)_ = 21.76, *p* < 0.001, Stress *F*_(1, 74)_ = 15.59, *p* < 0.001, Genotype × Stress *F*_(2, 74)_ = 3.22, *p* = 0.045; **No Stress**: *Drd2*^+/+^ vs. *Drd2*^+/−^*p* = 0.016, *Drd2*^+/+^ vs. *Drd2*^−/−^*p* = 0.001; **CMS**: *Drd2*^+/+^ vs. *Drd2*^−/−^*p* < 0.001, *Drd2*^+/−^ vs. *Drd2*^−/−^*p* = 0.003; ***Drd2***^+/−^: CMS vs. No Stress: *p* = 0.002. **DM striatum**: Genotype *F*_(2, 74)_ = 14.14, *p* < 0.001, Stress *F*_(1, 74)_ = 12.36, *p* < 0.001, Genotype × Stress *F*_(2, 74)_ = 4.87, *p* = 0.010; **No Stress**: *Drd2*^+/+^ vs. *Drd2*^+/−^*p* = 0.007, *Drd2*^+/+^ vs. *Drd2*^−/−^*p* = 0.013; **CMS**: *Drd2*^+/+^ vs. *Drd2*^−/−^*p* = 0.006, *Drd2*^+/−^ vs. *Drd2*^−/−^*p* = 0.003; ***Drd2***^+/−^: CMS vs. No Stress: *p* < 0.001. **VM striatum**: Genotype *F*_(2, 74)_ = 19.45, *p* < 0.001, Stress *F*_(1, 74)_ = 15.21, *p* < 0.001, Genotype × Stress *F*_(2, 74)_ = 4.01, *p* = 0.022; **No Stress**: *Drd2*^+/+^ vs. *Drd2*^+/−^*p* = 0.005, *Drd2*^+/+^ vs. *Drd2*^−/−^*p* = 0.002; **CMS**: *Drd2*^+/+^ vs. *Drd2*^−/−^*p* < 0.001, *Drd2*^+/−^ vs. *Drd2*^−/−^*p* = 0.002; ***Drd2***^+/−^: CMS vs. No Stress: *p* < 0.001. **Nucleus Accumbens**: Genotype *F*_(2, 74)_ = 5.54, *p* = 0.005, Stress *F*_(1, 74)_ = 14.17, *p* < 0.001, Genotype × Stress *F*_(2, 74)_ = 4.49, *p* = 0.014; **CMS**: *Drd2*^+/−^ vs. *Drd2*^−/−^*p* = 0.04; ***Drd2***^+/−^: CMS vs. No Stress: *p* < 0.001. **Olfactory tubercle**: Genotype *F*_(2, 74)_ = 6.74, *p* = 0.002, Stress *F*_(1, 74)_ = 9.46, *p* = 0.003. **D tail**: Genotype *F*_(2, 74)_ = 10.45, *p* < 0.001, Stress *F*_(1, 74)_ = 15.22, *p* < 0.001, Genotype × Stress *F*_(2, 74)_ = 3.47, *p* = 0.036; **CMS**: *Drd2*^+/+^ vs. *Drd2*^−/−^*p* = 0.023, *Drd2*^+/−^ vs. *Drd2*^−/−^*p* = 0.001; ***Drd2***^+/−^: CMS vs. No Stress: *p* < 0.001. **V tail**: Genotype *F*_(2, 74)_ = 17.99, *p* < 0.001, Stress *F*_(1, 74)_ = 19.04, *p* < 0.001, Genotype × Stress *F*_(2, 74)_ = 4.47, *p* = 0.036; **CMS**: *Drd2*^+/+^ vs. *Drd2*^−/−^*p* = 0.006, *Drd2*^+/−^ vs. *Drd2*^−/−^*p* < 0.001; ***Drd2***^+/−^: CMS vs. No Stress: *p* = 0.001.).

### D2 receptor binding

**Figure 2:**
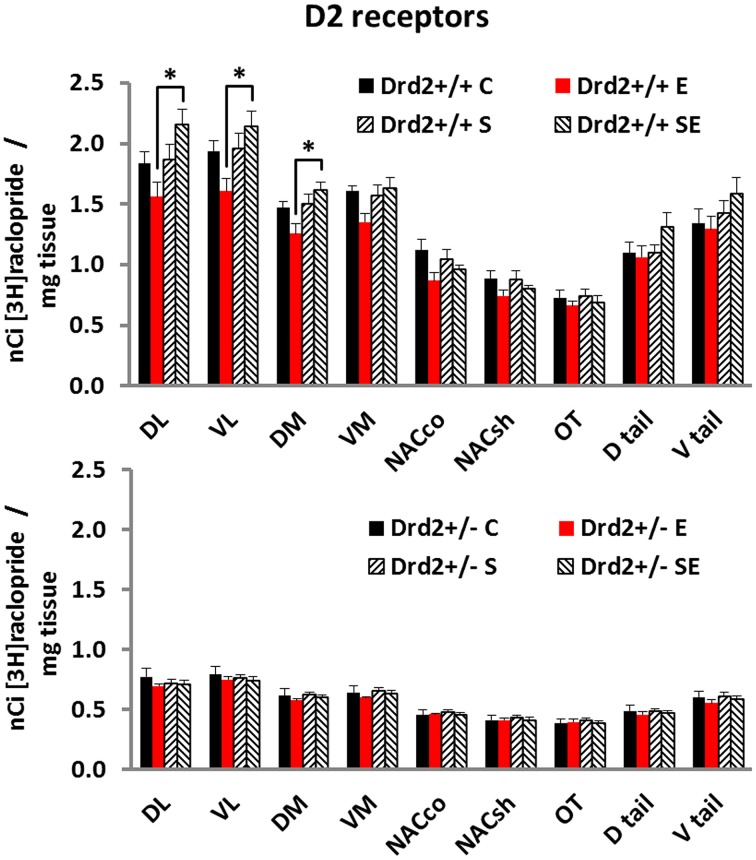
**[^3^H]Raclopride specific binding in the basal forebrain of *Drd2*^+/+^ and *Drd2*^+/−^mice**. The bars represent mean + S.E.M.; C, control; E, ethanol; S, chronic mild stress; SE, chronic mild stress and ethanol; *Drd2*^+/−^ measures are significantly different from the respective *Drd2*^+/+^ measures (for details please see Results).

We also studied changes in the basal forebrain D2 receptor levels, as determined with [^3^H]raclopride specific binding, associated with *Drd2* expression, with CMS exposure, and with ETOH intake (Figure 2). Three-way ANOVA for [^3^H]raclopride specific binding showed a significant main effect of Genotype in all regions studied and a Genotype × Stress × Ethanol interaction in the DL, VL, and DM quadrants of the striatum. Tukey *post-hoc* tests showed that *Drd2*^+/+^ mice had significantly higher D2 dopamine receptor levels compared with *Drd2*^+/−^ mice in all regions studied. The results also showed that *Drd2*^+/+^ ES mice had significantly higher D2 receptor levels than *Drd2*^+/+^ E mice in the DL, VL, and DM quadrants of the striatum (**DL striatum**: Genotype *F*_(1, 52)_ = 342.71, *p* < 0.001, Genotype × Stress × Ethanol *F*_(1, 52)_ = 4.34, *p* = 0.042; **Genotype**: *Drd2*^+/+^ C vs. *Drd2*^+/−^ C *p* < 0.001, *Drd2*^+/+^E vs. *Drd2*^+/−^ E *p* < 0.001, *Drd2*^+/+^S vs. *Drd2*^+/−^ S *p* < 0.001, *Drd2*^+/+^ES vs. *Drd2*^+/−^ ES *p* < 0.001; ***Drd2***^+/+^: E vs. ES *p* < 0.001. **VL striatum**: Genotype *F*_(1, 52)_ = 360.73, *p* < 0.001, Genotype × Stress × Ethanol *F*_(1, 52)_ = 4.30, *p* = 0.043; **Genotype**: *Drd2*^+/+^ C vs. *Drd2*^+/−^ C *p* < 0.001, *Drd2*^+/+^E vs. *Drd2*^+/−^ E *p* < 0.001, *Drd2*^+/+^S vs. *Drd2*^+/−^ S *p* < 0.001, *Drd2*^+/+^ES vs. *Drd2*^+/−^ ES *p* < 0.001; ***Drd2***^+/+^: E vs. ES *p* = 0.002. **DM striatum**: Genotype *F*_(1, 52)_ = 485.75, *p* < 0.001, Genotype × Stress × Ethanol *F*_(1, 52)_ = 4.03, *p* = 0.047; **Genotype**: *Drd2*^+/+^ vs. *Drd2*^+/−^ *p* < 0.001, *Drd2*^+/+^E vs. *Drd2*^+/−^ E *p* < 0.001, *Drd2*^+/+^S vs. *Drd2*^+/−^ S *p* < 0.001, *Drd2*^+/+^ES vs. *Drd2*^+/−^ ES *p* < 0.001; ***Drd2***^+/+^: E vs. ES *p* = 0.001. **VM striatum**: Genotype *F*_(1, 52)_ = 467.42, *p* < 0.001; *Drd2*^+/+^ vs. *Drd2*^+/−^ *p* < 0.001. **Nucleus accumbens core**: Genotype *F*_(1, 52)_ = 191.51, *p* < 0.001; *Drd2*^+/+^ vs. *Drd2*^+/−^ *p* < 0.001. **Nucleus accumbens shell**: Genotype *F*_(1, 52)_ = 170.40, *p* < 0.001; *Drd2*^+/+^ vs. *Drd2*^+/−^ *p* < 0.001. **Olfactory tubercle**: Genotype *F*_(1, 52)_ = 109.67, *p* < 0.001; *Drd2*^+/+^ vs. *Drd2*^+/−^ *p* < 0.001. **Dtail**: Genotype *F*_(1, 52)_ = 200.53, *p* < 0.001; *Drd2*^+/+^ vs. *Drd2*^+/−^ *p* < 0.001. **Dtail**: Genotype *F*_(1, 52)_ = 192.29, *p* < 0.001; *Drd2*^+/+^ vs. *Drd2*^+/−^ *p* < 0.001).

### D1 receptor binding

**Figure 3:**
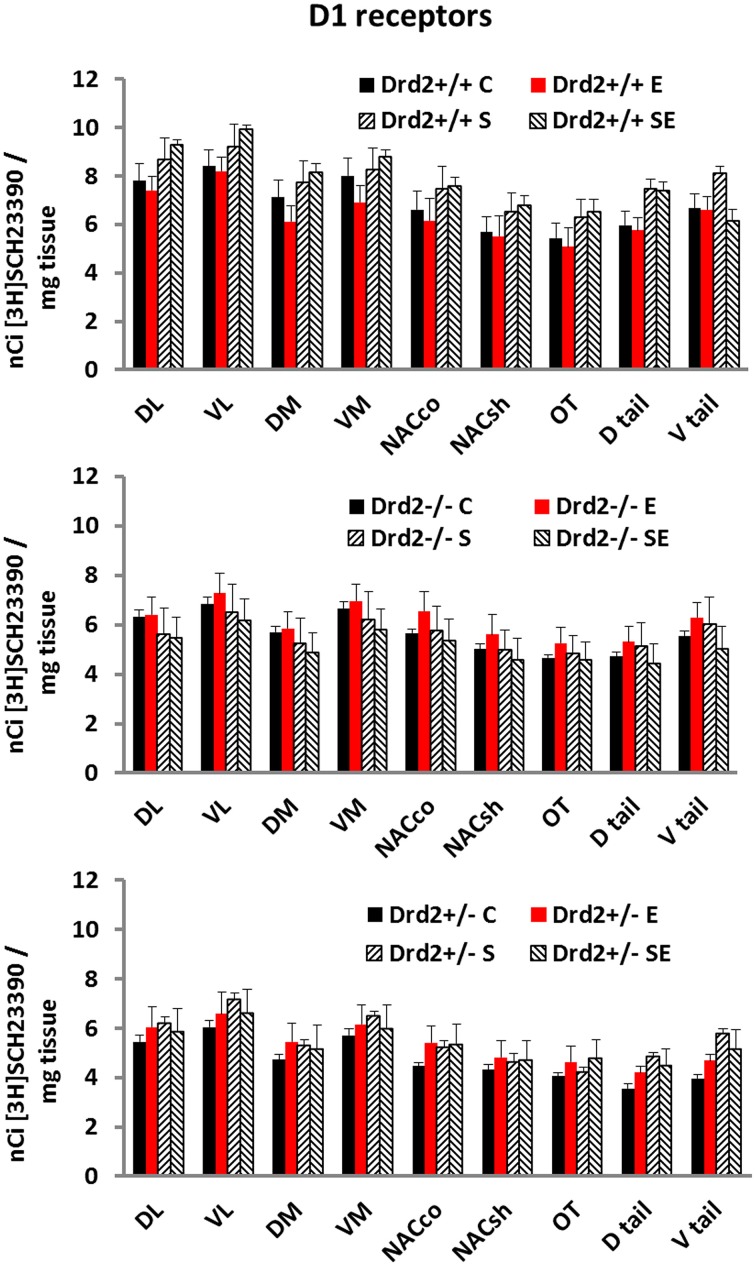
**[^3^H]SCH23390 specific binding in the basal forebrain of *Drd2*^+/+^, *Drd2*^+/−^, and *Drd2*^−/−^mice**. The bars represent mean + S.E.M.; C control, E ethanol, S chronic mild stress, SE chronic mild stress and ethanol; *Drd2*^+/−^ and *Drd2*^−/−^ measures are significantly different from the respective *Drd2*^+/+^ measures (for details please see Results).

The levels and distribution of D1 dopamine receptors in the basal forebrain, associated with *Drd2* expression, with exposure to CMS, and with ETOH intake, were studied with *in vitro* [^3^H]SCH23390 autoradiography (Figure 3). D1 dopamine receptor levels were lower in *Drd2*^+/−^ and *Drd2*^−/−^ mice, compared with *Drd2*^+/+^, in all regions examined (Three-way ANOVA, **DL striatum**: Genotype *F*_(2, 69)_ = 15.24, *p* < 0.001; *Drd2*^+/+^ vs. Drd2^+/−^*p* < 0.001, Drd2^+/+^ vs. Drd2^−/−^*p* < 0.001). **VL striatum**: Genotype *F*_(2, 69)_ = 13.36, *p* < 0.001; *Drd2*^+/+^ vs. Drd2^+/−^*p* < 0.001, Drd2^+/+^ vs. Drd2^−/−^*p* < 0.001). **DM striatum**: Genotype *F*_(2, 69)_ = 11.50, *p* < 0.001; *Drd2*^+/+^ vs. Drd2^+/−^*p* < 0.001, Drd2^+/+^ vs. Drd2^−/−^*p* = 0.002). **VM striatum**: Genotype *F*_(2, 69)_ = 8.16, *p* < 0.001; *Drd2*^+/+^ vs. Drd2^+/−^*p* = 0.001, Drd2^+/+^ vs. Drd2^−/−^*p* = 0.015). **Nucleus accumbens core**: Genotype *F*_(2, 69)_ = 6.64, *p* = 0.001; *Drd2*^+/+^ vs. Drd2^+/−^*p* = 0.002, Drd2^+/+^ vs. Drd2^−/−^*p* = 0.035). **Nucleus accumbens shell**: Genotype *F*_(2, 69)_ = 5.344, *p* = 0.007; *Drd2*^+/+^ vs. Drd2^+/−^*p* = 0.007, Drd2^+/+^ vs. Drd2^−/−^*p* = 0.039). **Olfactory tubercle**: Genotype *F*_(2, 69)_ = 5.325, *p* = 0.007; *Drd2*^+/+^ vs. Drd2^+/−^*p* = 0.007, Drd2^+/+^ vs. Drd2^−/−^*p* = 0.031). **Dtail**: Genotype *F*_(2, 69)_ = 21.75, *p* < 0.001; *Drd2*^+/+^ vs. Drd2^+/−^*p* = 0.007, Drd2^+/+^ vs. Drd2^−/−^*p* < 0.001). **Vtail**: Genotype *F*_(2, 69)_ = 13.96, *p* < 0.001; *Drd2*^+/+^ vs. Drd2^+/−^*p* < 0.001, Drd2^+/+^ vs. Drd2^−/−^*p* = 0.017).

### Correlations between receptor binding and ethanol intake

**Figure 4:**
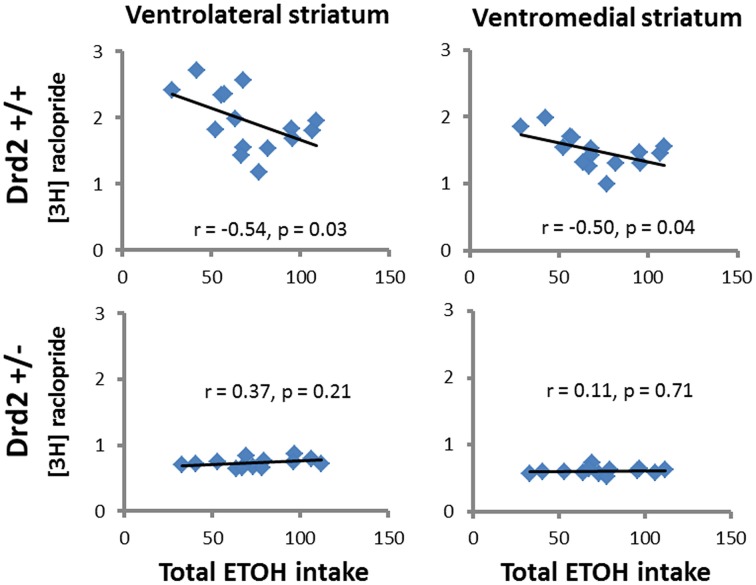
**Significant correlations between D2 receptor levels ([^3^H]raclopride specific binding, nCi/mg tissue) and total ethanol intake (g ETOH/kg b.w.) in *Drd2*^+/+^ and *Drd2*^+/−^ mice in the ventrolateral and ventromedial striatum**.

**Figure 5:**
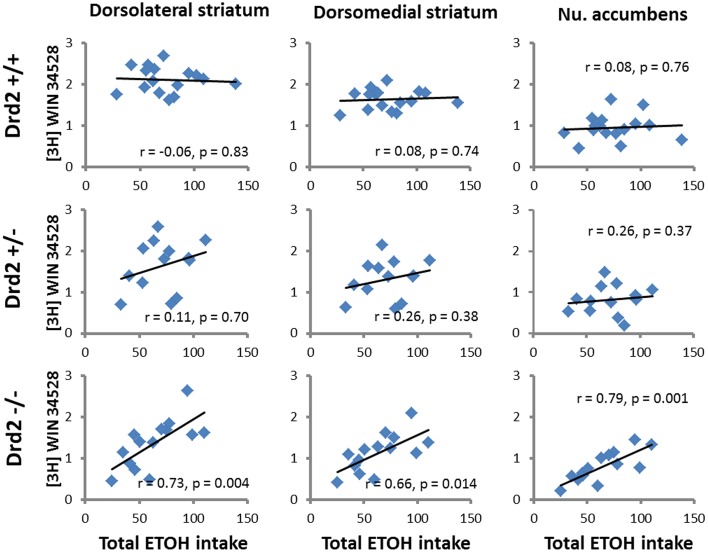
**Significant correlations between DAT levels ([^3^H]WIN35428 specific binding, nCi/mg tissue) and total ethanol intake (g ETOH/kg b.w.) in *Drd2*^+/+^, *Drd2*^+/−^, and *Drd2*^−/−^ mice in representative motor (dorsolateral striatum), cognitive (dorsomedial striatum), and limbic (nucleus accumbens) regions of the dopaminergic basal forebrain**.

ETOH intake in *Drd2*^+/+^ mice was negatively correlated with [^3^H]raclopride specific binding in the VM striatum (*r* = –0.54, *p* = 0.03) and the DL striatum (*r* = −0.5, *p* = 0.04) (Figure 4). Ethanol intake in *Drd2*^−/−^mice was positively correlated with [^3^H]WIN35428 specific binding in all regions studied (DL *r* = 0.73, *p* = 0.004; VL *r* = 0.76, *p* = 0.003; DM *r* = 0.657, *p* = 0.014; VM *r* = 0.722, *p* = 0.005; NAC *r* = 0.791, *p* = 0.001; OT *r* = 0.680, *p* = 0.011; Dtail *r* = 0.671, *p* = 0.012; Vtail *r* = 0.698, *p* = 0.008) (Figure 5).

## Discussion

**Figure 6:**
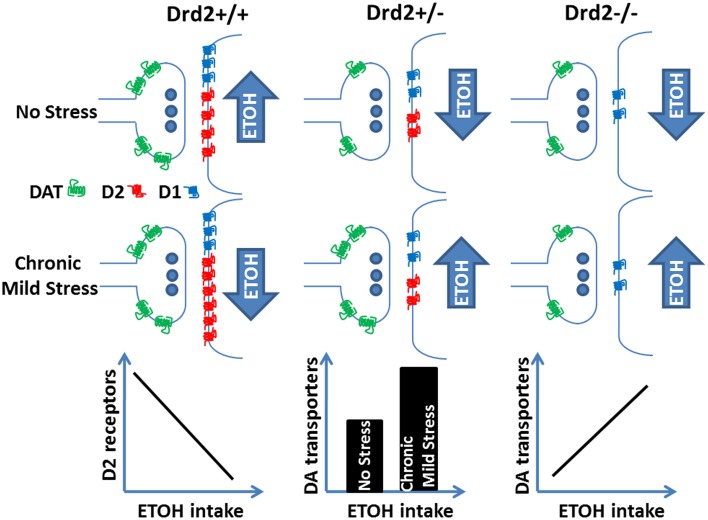
**Ethanol intake regulation by *Drd2* expression, dopamine receptor and transporter changes, and exposure to chronic mild stress**.

Overall, the current and our previous study (Delis et al., 2013) show that lower ETOH intake in *Drd2*^+/+^ mice exposed to 4 weeks of CMS is associated with higher D2 receptor levels in the striatum and that ETOH intake is negatively correlated with D2 receptor levels. In *Drd2*^+/−^ mice, D2 receptor levels are not affected by any treatment; perhaps their low expression levels do not allow for a modulatory role. In these mice, CMS exposure leads to higher ETOH intake and to higher DAT levels in the striatum and the n. accumbens. In *Drd2*^−/−^ mice, DAT levels do not change in response to the applied treatments, but they are positively correlated with ETOH intake. In addition, we show a significant decrease in D1 dopamine receptor levels throughout the dopaminergic basal forebrain of *Drd2*^+/−^ and *Drd2*^−/−^ mice, which is not affected by the applied treatments. Our findings on the regulation of ethanol consumption by CMS, *Drd2* expression, and markers of dopamine neurotransmission are schematically presented in Figure 6.

### Drd2 knockout decreases DAT and dopamine D1 receptor levels

*Drd2*^+/−^ and ^−/−^ mice have significantly lower DAT levels in the dopaminergic basal forebrain, compared with *Drd2*^+/+^ mice. Previous studies have shown that the lack of dopamine D2 receptors, which include the presynaptic D2 autoreceptors and the post-synaptic D2 receptors, leads to increased stimulus-induced DA release, increased extracellular DA half-life, lower DA clearance, and lower DA uptake (Dickinson et al., 1999; Rouge-Pont et al., 2002; Benoit-Marand et al., 2011). Our finding of lower DAT levels in *Drd2*^−/−^ mice suggests that the lack of *Drd2* expression leads to slower clearance and lower DA uptake *in vivo*, not only because of disinhibited DA release, but also because of a decrease in DAT levels which, in turn, could be a direct result of the disrupted DAT - D2 autoreceptor interaction (Lee et al., 2007).

Our finding of lower D1 receptor levels in *Drd2*^+/−^ and *Drd2*^−/−^ mice, compared with Drd2^+/+^, is in agreement with previous receptor binding and early gene expression studies showing lower D1 receptor binding (Short et al., 2006) and blunted D1-mediated c-fos expression in *Drd2*^−/−^ mice (Schmauss et al., 2002).

### Lower dopamine D1 receptor and DAT levels are associated with lower ETOH preference

Studies show that ETOH intake/preference is negatively associated with dopamine D2 receptor levels (Stefanini et al., 1992; McBride et al., 1993; Volkow et al., 1996; Thanos et al., 2001, 2004, 2005), which predicts that *Drd2*^+/−^ and *Drd2*^−/−^ mice would have higher ethanol intake/preference, compared with *Drd2*^+/+^. This, however, is not the case (Phillips et al., 1998; Delis et al., 2013). The paradoxical finding of lower ETOH intake and preference in *Drd2*^+/−^ and *Drd2*^−/−^ mice, compared with *Drd2*^+/+^, has been explained by the lack of reward-mediating D2 receptors. An additional plausible explanation is suggested by the current study and involves the lower D1 receptor levels in *Drd2*^+/−^ and *Drd2*^−/−^ C mice. D1 receptor antagonism prevents the reward potentiating effects of alcohol in the intracranial self-stimulation paradigm (Fish et al., 2014) and decreases ETOH seeking as well as ETOH conditioned place preference (Liu and Weiss, 2002; Hamlin et al., 2007; Chaudhri et al., 2009; Bahi and Dreyer, 2012; Pina and Cunningham, 2014; Sciascia et al., 2014; Young et al., 2014), while D1 agonism increases ETOH self-administration (D−souza et al., 2003) and ethanol-induced motor sensitization (Abrahao et al., 2011). Since the rewarding properties of ETOH are decreased by D1 receptor antagonism, we conclude that the lower D1 striatal receptor levels in *Drd2*^+/−^ and *Drd2*^−/−^ C mice contribute to their lower ETOH intake/preference, compared with *Drd2*^+/+^ mice.

Our finding of lower DAT levels in the dopaminergic basal forebrain of the non-preferring *Drd2*^+/−^ and *Drd2*^−/−^ C mice is in agreement with the lower ETOH intake levels in DAT^−/−^ mice, compared with DAT^+/+^ (Savelieva et al., 2002; Mittleman et al., 2011) (but see Hall et al., 2003) and complementary to findings of higher dopamine uptake and higher DAT levels in alcohol preferring HAD rats (Carroll et al., 2006) and ethanol preferring monkeys (Mash et al., 1996). On the other hand, studies show that ethanol preferring Sardinian rats have lower DAT levels (Casu et al., 2002) and that DAT levels fluctuate periodically as a function of the duration of ETOH exposure (Hamdi and Prasad, 1991). Studies in humans suggest that alcoholism, particularly Cloninger type I, is associated with lower DAT levels in the dorsal rather than the ventral striatum (Dobashi et al., 1997; Repo et al., 1999; Tupala et al., 2001), although other studies are often inconclusive (Xu and Lin, 2011) or show DAT associations with other aspects of alcohol addiction, such as visual and somatosensory hallucinations (Limosin et al., 2004; Huber et al., 2007), the severity of withdrawal (Schmidt et al., 1998; Gorwood et al., 2003), and novelty seeking (Bau et al., 2001; Laine et al., 2001) but not ETOH intake *per se*. It is obvious that the role of DAT in the regulation of ETOH preference remains inconclusive, particularly in humans. The findings of this study are in line with a majority of preclinical studies and suggest that in the *Drd2* genetic model of ETOH intake, lower DAT levels contribute to lower levels of ETOH intake.

### ETOH intake in CMS-exposed normal mice is moderated by D2 receptors

In this study we show that among ETOH consuming *Drd2*^+/+^ mice, those exposed to CMS have significantly higher D2 receptor levels in regions of the motor (lateral) and cognitive (dorsomedial) striatum. CMS-exposed *Drd2*^+/+^ mice have lower ETOH intake compared to non-CMS exposed *Drd2*^+/+^ mice (Delis et al., 2013), which suggests that high D2 receptor levels moderate ETOH intake, in agreement with previous studies in rodents and humans. Ethanol preferring rats express low D2 receptor levels (Stefanini et al., 1992; McBride et al., 1993; Thanos et al., 2001) in agreement with studies in humans showing that alcoholism is associated with lower post mortem and *in vivo* D2 receptors (Blum et al., 1990; Volkow et al., 1996; Tupala et al., 2001). In addition, *Drd2* overexpression decreases ETOH intake and preference in ETOH preferring and non-preferring rodents (Thanos et al., 2001, 2004, 2005), which is in agreement with studies in humans showing higher D2 receptor levels in unaffected members of families with alcoholics (Volkow et al., 2006). Our finding of a negative correlation between ETOH intake and D2 receptor levels in all ETOH consuming *Drd2*^+/+^ mice is in agreement with the aforementioned studies and supports the protective role of D2 receptors against ETOH overconsumption.

The lower ETOH intake levels in stressed *Drd2*^+/+^ mice were, perhaps, the most intriguing finding of our previous study (Delis et al., 2013). Instead of increasing ETOH intake after CMS exposure, as would be suggested if ethanol consumption was a self-medication strategy to counteract stress, the mice decreased consumption. Similar findings of lower ethanol consumption when stress and ethanol are experienced together or in proximity have been previously presented (Rockman and Glavin, 1986; van Erp and Miczek, 2001; van Erp et al., 2001; Chester et al., 2004; Clark et al., 2007; Deehan et al., 2011; Norman et al., 2014). A likely explanation of our result is that the stressors, in spite of being chronic and incontrollable, were presented to the mice in the same order each week, which may have allowed them to cope with stress (Miller, 1981; Koolhaas et al., 2011; Herman, 2013; Lucas et al., 2014). This would also explain the lack of effect of CMS on D2 receptors in *Drd2*^+/+^ mice, in agreement with studies showing normal D2 receptor levels in CMS-resilient rats (Zurawek et al., 2013). In agreement with this hypothesis, DRD2 antagonism or knockout impair cognitive function (Glickstein et al., 2002; Tillerson et al., 2006; Watson et al., 2012), which could account for the presumed difficulty of the *Drd2*^+/−^ and *Drd2*^−/−^ mice to predict the CMS stressors.

### DAT levels increase after CMS and remain high in ETOH-consuming CMS-exposed Drd2^+/−^ mice

In this study we observe a significant increase in DAT levels in CMS-exposed *Drd2*^+/−^ mice (S and SE), in most regions of the dopaminergic basal forebrain studied, compared with *Drd2*^+/−^ animals that were not exposed to CMS (C and E). Our finding is in agreement with previous preclinical studies showing higher DAT levels and function after exposure to various types of stress. DAT levels/function increase after chronic restraint stress (Copeland et al., 2005), immobilization stress (Lucas et al., 2007), social defeat (Novick et al., 2011), variable stress (Kohut et al., 2012), prenatal stress (Converse et al., 2013), and isolation rearing (Yorgason et al., 2013). In humans, the *DAT* gene contains a 40 nucleotide repeat polymorphism in the 3′ non-coding region that modulates the expression of the gene. This polymorphism may present with a variable number of repeats (R), with the 9R and 10R being the most frequent. The 9R polymorphism is particularly interesting in humans since its presence is associated with increased DAT expression levels (Michelhaugh et al., 2001; van de Giessen et al., 2009; Faraone et al., 2014) as well as post-traumatic stress disorder (Fuke et al., 2001; Segman et al., 2002; Valente et al., 2011; Hoexter et al., 2012). Therefore, preclinical and clinical findings converge to the suggestion that exposure to stress, including chronic mild stress, leads to increased DAT levels in the dopaminergic basal forebrain, in agreement with our findings of higher DAT levels in *Drd2*^+/−^ mice exposed to CMS. In addition, in humans, the high DAT expression levels induced by the 9R polymorphism are associated with novelty seeking in alcoholics (Bau et al., 2001; Laine et al., 2001) and with severe alcohol withdrawal symptomatology (Schmidt et al., 1998; Gorwood et al., 2003). In animals, high DAT levels are associated with chronic ethanol consumption in HAD rats (Carroll et al., 2006) and are markers of high ethanol preference in primates (Mash et al., 1996). Based on the above, we postulate that CMS-induced increases in DAT levels contribute to the higher ETOH intake observed in *Drd2*^+/−^ SE mice. Finally, although DAT levels do not change significantly between treatment groups in *Drd2*^−/−^ mice, they are positively correlated with ETOH intake, which is in the same line with our findings in *Drd2*^+/−^ mice and suggests that in the absence of *Drd2* expression, ETOH intake is primarily and positively regulated by DAT expression levels.

## Conclusions

The present study shows that in normal mice, ETOH intake is primarily, and negatively, regulated by dopamine D2 receptors. ETOH overconsumption under CMS is prevented in normal mice expressing high dopamine D2 receptor levels. The study also suggests that in mice with limited *Drd2* expression, ETOH overconsumption is prevented by lower D1 receptor and DAT levels. In these mice, ETOH intake under CMS is positively regulated by DAT. The existence of a regulatory role of DAT on ETOH intake is also evident in mice with no *Drd2* expression, in which DAT levels are positively correlated with ETOH intake. These findings illustrate the significant interaction between an environmental factor -stress- and *Drd2* and *Dat*, two fundamental genetic factors in the regulation of reward perception and handling (Comings and Blum, 2000; Blum et al., 2011, 2014). Our study is in agreement with previous findings on the role of D2 receptors in ETOH intake and reveals new mechanisms of ETOH intake regulation by D1 and DAT. Studies of neurotransmitter systems that interact with the dopaminergic system and that are involved in ETOH reward and addiction, such as the GABAergic and endocannabinoid systems, are necessary in order to further comprehend the biological mechanisms underlying the complex interactions between stress, brain, and ETOH consumption.

## Author contribution

All authors have contributed in the design, execution, analysis, and writing of this study.

### Conflict of interest statement

The authors declare that the research was conducted in the absence of any commercial or financial relationships that could be construed as a potential conflict of interest.
